# Maternal *Ureaplasma/Mycoplasma* colonization during pregnancy and neurodevelopmental outcomes for preterm infants

**DOI:** 10.3389/fped.2022.893812

**Published:** 2022-08-15

**Authors:** Francesca Gallini, Domenico Umberto De Rose, Maria Coppola, Maria Sofia Pelosi, Francesco Cota, Anthea Bottoni, Daniela Ricci, Domenico Marco Romeo, Teresa Spanu, Luca Maggio, Eugenio Mercuri, Giovanni Vento

**Affiliations:** ^1^Neonatology Unit, Department of Woman and Child Health and Public Health, Fondazione Policlinico Universitario “Agostino Gemelli” Istituto di Ricovero e Cura a Carattere Scientifico, Rome, Italy; ^2^Università Cattolica del Sacro Cuore, Milan, Italy; ^3^Neonatal Intensive Care Unit, Medical and Surgical Department of Fetus—Newborn—Infant, “Bambino Gesù” Children’s Hospital Istituto di Ricovero e Cura a Carattere Scientifico, Rome, Italy; ^4^National Centre of Services and Research for the Prevention of Blindness and Rehabilitation of Low Vision Patients–International Agency for the Prevention of Blindness (IAPB) Italia Onlus, Rome, Italy; ^5^Pediatric Neurology Unit, Department of Woman and Child Health and Public Health, Fondazione Policlinico Universitario “Agostino Gemelli” Istituto di Ricovero e Cura a Carattere Scientifico, Rome, Italy; ^6^Department of Laboratory and Infectious Sciences, Fondazione Policlinico Universitario “Agostino Gemelli” Istituto di Ricovero e Cura a Carattere Scientifico, Rome, Italy; ^7^Neonatology Unit, S. Camillo—Forlanini Hospital, Rome, Italy

**Keywords:** neonate, newborn, neurodevelopment, cognitive, pregnant, prematurity—risk assessment and prevention, motor performance, motor outcomes

## Abstract

**Introduction:**

*Ureaplasma* (*U*.) and *Mycoplasma* (*M*.) species have been related to pregnancy complications (including preterm birth) and worse neonatal outcomes. The aim of our work is to evaluate neurodevelopmental outcomes in preterm infants born to mothers with *Ureaplasma*/*Mycoplasma* colonization during pregnancy.

**Methods:**

Preterm infants with gestational age (GA) of ≤ 30 weeks were included in a retrospective follow-up study. To evaluate the effects of maternal vaginal colonization, we divided preterm infants into two groups: exposed and unexposed infants. All infants were assessed at 24 ± 3 months of age using Griffith’s Mental Developmental Scales (GMDS).

**Results:**

Among 254 preterm infants, only 32 infants (12.6%) were exposed to U. /M. colonization during pregnancy. Exposed infants and unexposed ones had a similar Griffith′s Developmental Quotient (106 ± 27.2 vs. 108.9 ± 19.5, respectively), without significant differences (*p* = 0.46). However, exposed infants had a significantly poorer outcome than their unexposed peers in terms of locomotor abilities (100.7 ± 28.3 exposed vs. 111.5 ± 26.1 unexposed, *p* = 0.03).

**Conclusion:**

For visual and hearing impairment, exposed and unexposed infants had similar incidences of cognitive and motor impairment. However, exposed infants had significantly lower locomotor scores than unexposed peers.

## Introduction

*Ureaplasma (U.)* and *Mycoplasma (M.)* species have been related to pregnancy complications (including preterm birth) and worse neonatal outcomes (1, 2). The data from clinical and animal studies, as well as *in vitro* findings, suggest neuroinflammatory patterns in exposed mother/infant pairs (3).

Vaginal swabs can be used to quickly rule out bacterial colonization in mothers during pregnancy. However, given the high frequency of vaginal *Ureaplasma/Mycoplasma* spp. colonization in asymptomatic sexually active women, we still do not know the actual clinical impact of positive maternal swabs regarding neonatal outcomes (4–6).

Different authors investigated their concerns on *Ureaplasma*-driven neuroinflammation in neonates (3). Viscardi et al. described how the presence of *Ureaplasma* was significantly associated with elevated interleukin-1-beta in cord blood and *Ureaplasma* serum-positive infants had a 2.3-fold increased risk of severe intraventricular hemorrhage (IVH) (7). This has been confirmed by Kasper et al., not only for severe IVH, but also when including all the IVH grades (8). Isolation of *Ureaplasma* species from the amniotic cavity cultures at birth also resulted to be significantly associated with an abnormal psychomotor development index, an abnormal neurologic outcome, and a higher probability for diagnosis of cerebral palsy at 2 years of age when compared to patients with negative culture results, according to Berger’s findings (9).

The aim of our work was to evaluate neurodevelopmental outcomes in preterm infants born to mothers with *Ureaplasma/Mycoplasma* vaginal colonization during pregnancy.

## Materials and methods

### Design of the study

A total of 254 preterm infants with gestational age (GA) of ≤ 30 weeks (born in our hospital between June 2012 and June 2017) were enrolled in a previous retrospective study about the extra-uterine growth restriction and related neurodevelopmental outcomes, as previously published (10). All included infants regularly attended our follow-up program at least up to 24 months of corrected age (CA), as per our unit protocol (11), up to November 2019. Exclusion criteria were death after discharge, incomplete medical records, congenital malformations, genetic syndromes, large for gestational age (LGA) at birth, non-Italian speaker, lost to follow-up (not present at 2 sequential follow-up visits), and inclusion in any trial that could interfere with growth and outcomes (10). Data of patients were obtained from medical records.

**FIGURE 1:**
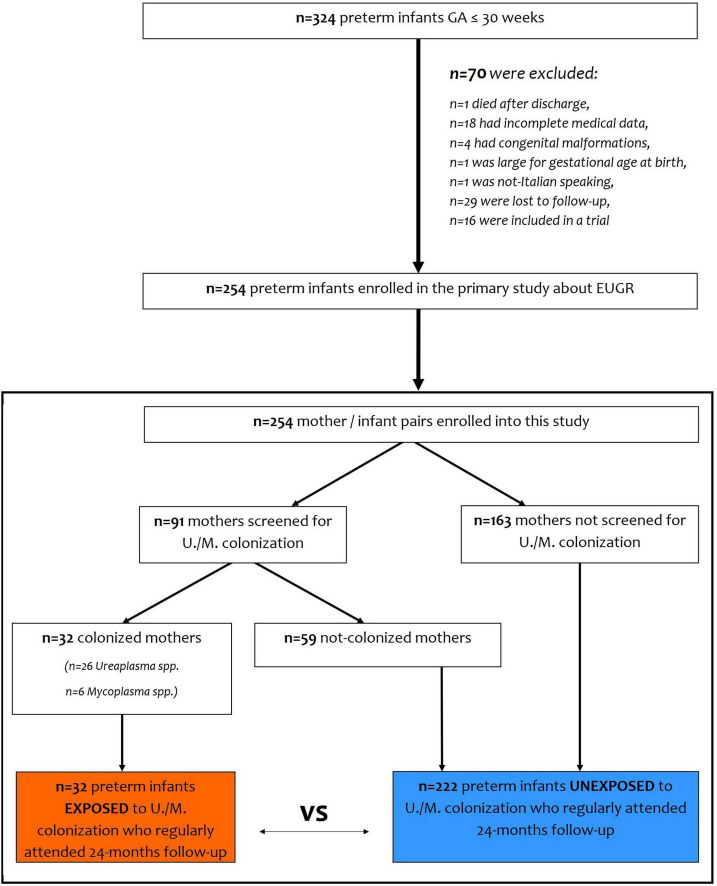
Flow-chart of the study design.

In order to evaluate the effects of maternal intrauterine inflammation, we divided preterm infants into two groups: infants born to mothers with *Ureaplasma*/*Mycoplasma* colonization during pregnancy (cases) and unexposed infants (controls). We also considered unexposed infants born to mothers who were not screened, and pregnancy was uneventful until delivery (Figure 1).

All mothers with the threat of miscarriage or preterm labor were screened for *Ureaplasma/Mycoplasma* colonization; samples from the lower genital tract were obtained using vaginal swabs during physical examinations. Colonization was diagnosed *via* microbial culture or DNA extraction and PCR analysis according to diagnostic conditions employed at the microbiology unit of each laboratory where the mothers went.

All exposed preterm infants received clarithromycin in the first 3 days of life, after baseline respiratory specimens (nasal swabs if in spontaneous breathing or in non-invasive ventilation; bronchoalveolar lavage fluid if intubated) were collected. In our hospital, culture and identification were performed until 2015 using the *Mycoplasma* IST 2 kit (BioMérieux, Craponne, France), and we were able to identify *U. urealyticum* and *M. hominis* (12). Afterward, the method used has been changed into the new molecular Anyplex™ STI-7 Detection kit (Seegene Inc., Seoul, Korea), based on a multiplex real-time PCR method that can identify *M. genitalium*, *M. hominis*, *U. parvum*, and *U. urealyticum* (13).

Antibiotic treatment was stopped when samples resulted negative.

### Neonatal outcomes

Data were collected in our follow-up facility care unit, including pregnancy and newborn characteristics.

The following maternal and neonatal characteristics were collected during the neonatal period: maternal age, antenatal corticosteroids, complete course of antenatal corticosteroids, multiple pregnancy, pathological umbilical artery Doppler parameters, cesarean section, preterm premature rupture of membranes, sex of the neonate, GA, birthweight, birth head circumference, Apgar at 5 min, number of days on invasive mechanical ventilation, incidence of bronchopulmonary dysplasia (BPD, defined by the need for supplemental oxygen at 36 w of PMA), postnatal steroids, presence of major brain lesions [defined as a grade ≥ III Intraventricular Hemorrhage (IVH) according to Papile or a Cystic Periventricular Leukomalacia (cPVL) according to De Vries (14, 15)], incidence of necrotizing enterocolitis (NEC, defined as a stage ≥ IIA according to Bell’s criteria), incidence of early-onset sepsis (EOS, defined as the presence of systemic signs suggestive of infection and positive blood and/or cerebrospinal fluid (CSF) culture before 72 h from birth), incidence of late-onset sepsis (LOS, defined as the presence of systemic signs suggestive of infection and positive blood and/or CSF culture after 72 h from birth), incidence of hemodynamically significant patent ductus arteriosus (PDA, i.e., pharmacologically or surgically treated), and days of parenteral nutrition.

### Long-term outcomes

All infants were assessed at 24 ± 3 months of age by an expert pediatric neurologist using Italian-validated translation of Griffith′s Mental Developmental Scales (GMDS) (0–2 years) (16).

GMDS yields five subscales: Locomotor, Personal-Social, Hearing and Speech, Eye and Hand Coordination, and Performance. The subscales yield standardized scores for each domain and a composite developmental quotient. The cognitive developmental outcome was classified normal when Griffiths’ developmental quotient (GDQ) was > 85; borderline, when GDQ was from 70 to 85; and delayed when GDQ was less than 70 (17).

Conversely, the motor outcome was classified as normal development, “minor neurological dysfunction” (MND, according to Touwen) (18), and cerebral palsy (CP, according to Bax) (19).

The onset of epilepsy was also recorded.

The severity of Retinopathy of prematurity (ROP) was defined as stage of ≥ 3, according to ICROP criteria (20), whereas all infants underwent an Auditory Brain Response test at 3 months CA to identify hearing impairment. Mild hearing loss was defined as the auditory threshold of 15–40 dB, moderate hearing loss if 40–70 dB, severe if 70–90 dB, and deafness if > 90 dB (21).

Disability was classified according to a previous scheme utilized in the EPICure studies (22), classifying outcomes as severe, moderate, and mild or no impairment using defined categories in motor, developmental, sensory, and communication domains.

### Statistical analysis and ethical approval

Data are presented as numbers and percentages for categorical variables. Continuous variables are expressed as mean ± standard deviation (SD) if they were normally distributed or as median and interquartile range if normality could not be accepted. Categorical variables were compared using the Fisher’s exact test or Chi-square with Yates correction. *T*-test was used to compare cases and controls in terms of Griffiths’ development quotient (GDQ) and neurodevelopment impairment (NDI). A *p*-value < 0.05 was considered significant.

Multivariate analysis (by means of logistic regression in case of binary outcome or linear regression in case of continuous variable outcome) was considered when appropriate, after the correction by GA and the presence of major brain lesions (IVH ≥ III grade or cPVL).

Statistical analysis was performed using software programs Microsoft Excel (2016 for Windows) and STATA/IC (version 15.1 for Windows).

The study was carried out in compliance with the Declaration of Helsinki and its later amendments, approved by the Ethics Committee of Fondazione Policlinico Universitario “Agostino Gemelli,” Rome, Italy (in the context of the protocol number 0036181/20 - ID 3244), and written informed consent was obtained from parents for any clinical research purpose about clinical data.

### Results

Maternal characteristics and neonatal characteristics of the study patients are reported in Table 1. Among 254 preterm infants, only thirty-two infants (12.6%) were reported to be exposed to *U./M*. colonization during pregnancy: among these, 26 were exposed to *Ureaplasma* (10 *U. urealyticum*, 4 *U. parvum*, 12 *U.* spp. Not specified) and 6 were exposed to *Mycoplasma* spp. *Mycoplasma* cases were not typed.

**TABLE 1:** Maternal characteristics and neonatal characteristics at birth and during NICU stay.

	Whole cohort (*n* = 254)	*Ureaplasma/Mycoplasma* exposed infants (*n* = 32)	*Ureaplasma/Mycoplasma* unexposed infants (*n* = 222)	*P*-value
**Maternal characteristics**				
Maternal age (years), mean ± *SD* (range)	34.2 ± 5.9	36.2 ± 5.6	34.2 ± 5.9	0.07
Antenatal corticosteroids, *n* (%)	221 (87.0)	27 (84.4)	194 (87.4)	0.58
Complete course of antenatal corticosteroids, *n* (%)	113 (44.5)	22 (68.8)	91 (41.0)	**<0.01**
Multiple birth, *n* (%)	82 (32.3)	2 (6.3)	80 (36.0)	**<0.01**
Pathological umbilical artery Doppler parameters, *n* (%)	55 (21.7)	5 (15.6)	50 (22.5)	1.00
Cesarean section, *n* (%)	214 (84.3)	23 (71.9)	191 (86.0)	0.06
Preterm premature rupture of membranes, *n* (%)	104 (40.9)	18 (56.3)	86 (38.7)	0.08
**Neonatal characteristics**				
Males, *n* (%)	126 (49.6)	19 (59.4)	107 (48.2)	1.00
Gestational age (weeks), mean ± *SD* (range)	28.6 ± 1.5 (23.5–30.6)	28.1 ± 1.7 (25.1–30.4)	28.4 ± 1.5 (23.5–30.6)	0.30
Birthweight (grams), mean ± *SD* (range)	1,090 ± 283 (500–1,820)	1,124 ± 272 (610–1,680)	1,095 ± 288 (500–1820)	0.59
Birth head circumference (centimeters), mean ± *SD*	25.9 + 2.1	25.7 ± 2.3	25.4 ± 2.0	0.44
Apgar at 5 min, mean ± *SD*	8.4 ± 0.9	8.4 ± 1.1	8.4 ± 0.8	1.00
Number of days on invasive mechanical ventilation, mean ± *SD*	6.7 ± 36.4	5.3 ± 11.6	4.3 ± 10.8	0.63
BPD at 36 weeks, *n* (%)	21 (8.3)	3 (9.4)	18 (8.1)	0.74
Postnatal steroids, *n* (%)	21 (8.3)	4 (12.5)	17 (7.7)	0.32
Patients with major brain lesions, *n* (%)	20 (7.9)	4 (12.5)	16 (7.2)	0.29
ROP ≥ 3, *n* (%)	6 (2.4)	0	6 (2.7)	0.75
NEC ≥ 2, *n* (%)	11 (4.3)	1 (3.1)	10 (4.5)	1.00
Early-onset Sepsis, *n* (%)	15 (5.9)	3 (9.4)	12 (5.4)	0.41
Late-onset Sepsis, *n* (%)	92 (36.2)	8 (25)	84 (37.8)	0.17
Hemodynamically Significant PDA, *n* (%)	65 (25.6)	5 (15.6)	60 (27.0)	0.27
Number of days of parenteral nutrition, mean ± *SD*	22.5 ± 17.6	18.4 ± 14.7	22.8 ± 16.7	0.16

Bold values are those that resulted to be statistically significant.

However, only 91 mothers were screened for *U./M*. colonization. If we only consider them, the colonization rate increases up to 32/91 cases (64.8%).

Mean GA at the time of *U./M*. detection was 19.3 ± 7.3 weeks GA (range: 7–29). The two groups of exposed and unexposed infants were similar in all maternal characteristics, except for the administration of antenatal corticosteroids (significantly higher in the exposed group) and twin pregnancies (higher in the unexposed group).

None of the preterm infants had a respiratory culture obtained after birth that resulted positive for *U./M*. spp. Exposed infants had a higher incidence of EOS, but not statistically significant. Furthermore, we identified no cases of early-onset and late-onset sepsis due to *U./M*. spp.

In Table 2, we show how neurodevelopmental outcomes at 24 months CA were similar in exposed and unexposed infants to *U./M*. colonization during pregnancy.

**TABLE 2:** Neurodevelopmental outcomes at 24 months CA in exposed and unexposed infants to *U./M*. colonization during pregnancy.

	Whole cohort (*n* = 254)	*Ureaplasma/Mycoplasma* exposed infants (*n* = 32)	*Ureaplasma/Mycoplasma* unexposed infants (*n* = 222)	*P*-value
**Cognitive impairment at 24 months CA**				
No cognitive impairment, *n* (%)	231 (90.9)	27 (84.4)	204 (91.9)	0.18
Mild, *n* (%)	14 (5.5)	3 (9.4)	11 (5.0)	0.40
Moderate, *n* (%)	4 (1.6)	2 (6.3)	2 (0.9)	0.08
Severe, *n* (%)	5 (2.0)	0	5 (2.3)	0.86
**Motor impairment at 24 months CA**				
No motor impairment, *n* (%)	207 (81.5)	28 (87.5)	179 (80.6)	0.47
MND, *n* (%)	26 (10.2)	1 (3.1)	25 (11.3)	0.22
Ambulatory cerebral palsy, *n* (%)	13 (5.1)	1 (3.1)	12 (5.4)	0.91
Not ambulatory cerebral palsy, *n* (%)	8 (3.1)	2 (6.3)	6 (2.7)	0.27
**Visual impairment at 24 months CA**				
No visual impairment, *n* (%)	225 (88.6)	31 (96.9)	194 (87.4)	0.14
Strabismus/refraction defects, *n* (%)	19 (7.5)	1 (3.1)	18 (8.1)	0.48
Hypovision, *n* (%)	10 (3.9)	0	10 (4.5)	0.62
Blindness, *n* (%)	0	0	0	1.00
**Hearing impairment at 24 months CA**				
No hearing impairment, *n* (%)	229 (90.2)	29 (90.6)	200 (90.1)	0.92
Mild (uncorrected), *n* (%)	12 (4.7)	2 (6.3)	10 (4.5)	0.65
Moderate (corrected with hearing aids), *n* (%)	11 (4.3)	1 (3.1)	10 (4.5)	0.74
Deep, *n* (%)	2 (0.8)	0	2 (0.9)	0.59
**Disability at 24 months CA**				
No, *n* (%)	207 (81.5%)	25 (78.1)	182 (82.0)	0.63
Mild, *n* (%)	23 (9.1)	4 (12.5)	19 (8.6)	0.51
Moderate, *n* (%)	11 (4.3)	1 (3.1)	10 (4.5)	0.72
Severe, *n* (%)	13 (5.1)	2 (6.3)	11 (5.0)	0.67

Indeed, exposed infants and unexposed ones had a similar GDQ (106 ± 27.2 vs. 108.9 ± 19.5, respectively), without significant differences (*p* = 0.46). Analyzing Griffith′s subscales, exposed infants had a significantly poorer outcome than their unexposed peers in terms of locomotor abilities (subscale A, 100.7 ± 28.3 exposed vs. 111.5 ± 26.1 unexposed, *p* = 0.03). Conversely, unexposed infants achieved similar results in other subscales: Personal-Social interactions (subscale B), Hearing and Language assessment (subscale C), Eye and Hand Coordination (subscale D), and Performance scale (subscale E) (Table 3). We tested the result of locomotor abilities with linear regression to verify if the effect of maternal colonization was independent of confounding factors. Maternal colonization was still significantly associated with the locomotor score after correcting with GA, IVH ≥ III grade, and cPLV (OR –10.3; CI –20.5/–0.04; *p* = 0.04).

**TABLE 3:** Griffiths’ at 24 months CA in exposed and unexposed infants to *U./M*. colonization during pregnancy.

	*Ureaplasma/Mycoplasma* exposed infants (*n* = 32)	*Ureaplasma/Mycoplasma* unexposed infants (*n* = 222)	*P*-value
Griffiths’ developmental quotient (GDQ)	106.0 ± 27.2	108.9 ± 19.5	0.46
**Griffiths’ subscales**			
**A:** Locomotor abilities	100.7 ± 28.3	111.5 ± 26.1	**0.03**
**B:** Personal-Social interactions	115.2 ± 25.3	116.1 ± 35.8	0.89
**C:** Hearing and Language assessment	98.5 ± 28.0	94.9 ± 33.2	0.56
**D:** Eye and Hand Coordination	101.9 ± 17.0	101.9 ± 25.1	1.00
**E:** Performance scale	117.2 ± 26.9	119.7 ± 35.2	0.70

Bold values are those that resulted to be statistically significant.

## Discussion

The detection of *Mycoplasma* and *Ureaplasma* spp. in vaginal cultures has been associated with spontaneous miscarriage, preterm birth, and chorioamnionitis (23). After preterm birth, perinatal *Ureaplasma* exposure might have a role in the development of neonatal inflammation, infection, and lung damage (24).

In this study we compared neonatal outcomes following maternal colonization during pregnancy due to *Mycoplasma* and *Ureaplasma*, comparing two groups with similar characteristics, in terms of GA and birthweight. We found no significant differences in the incidence of BPD at 36 w and other comorbidities, according to maternal colonization. However, the higher percentage of a complete course of antenatal steroids in the exposed group could have contributed to this finding.

The association of BPD with *Ureaplasma* spp., as reported by other studies, probably depends on a perinatal intrauterine infection (25) or a high-degree maternal colonization (≥10^4^ colony-changing units/ml) (26), rather than a single finding during pregnancy.

*Ureaplasma* spp. stimulate the release of tumor necrosis factor-alpha (TNFα), interleukin-1beta (IL-1β), interleukin-8 (IL-8), monocyte chemoattractant-1 (MCP-1), transforming growth factor-beta 1 (TGFβ1), and other mediators by various cell types *in vitro*, and *Ureaplasma* spp. colonization is associated with increased concentrations of these cytokines in tracheal aspirates during the first week of life in infants who develop BPD (27). Interleukin-6 (IL-6) stimulates the local antigen-specific immune response and especially exerts important anti-inflammatory effects in the lungs. By partially blocking the IL-6 response to lipopolysaccharide, *Ureaplasma urealyticum* might neutralize the downregulation of proinflammatory cytokines (28). The multiple banded antigen (MBA) is a surface lipoprotein that is the predominant pathogen-associated molecular pattern (PAMP) detected by the host immune system and has been proposed as the major *Ureaplasma* virulence factor (29).

*Ureaplasma* exposure can be the first “hit,” downregulating the host response, while microbial dysbiosis changes in the relative abundance of *Proteobacteria* and *Firmicutes*, and reduced *Lactobacilli* may be linked to the progression and severity of BPD (28). More research on microbiome optimization in preterm infants at risk for BPD is needed (30).

Exposed infants had a higher global incidence of early-onset sepsis from other microorganisms than *U./M*., although not significantly. This is in line with findings by Kasper et al., who found a relationship between the bacterial load of *Ureaplasma* in amniotic fluid and an increased intrauterine inflammatory response (31).

Concerning neurodevelopmental outcomes at 2 years, *Ureaplasma*-mediated brain injury is probably due to cytokine activation of the central nervous system immune response, with a fivefold increased risk for severe IVH in case of *Ureaplasma*-positive sera and increased serum IL-1β (32).

In our study, exposed and unexposed infants to maternal *Ureaplasma* during pregnancy had similar incidences of cognitive and motor impairment, as for visual and hearing impairment. We observed no differences in terms of disability. These findings are similar to those reported by Viscardi et al. who recently published the 2-year outcomes of a double-blind, placebo-controlled randomized trial of azithromycin to eradicate *Ureaplasma* respiratory colonization in preterm infants. They did not observe strong evidence of a difference in long-term neurodevelopment outcomes (assessed using the Bayley Scales of Infant and Toddler Development, third edition) in preterm infants treated with azithromycin in the first week of life compared to placebo (33).

However, we observed significantly lower scores in Griffith′s subscale concerning locomotor abilities in exposed infants. The biological plausibility of this result is based on *Ureaplasma*-driven systemic inflammation, well-confirmed *in vitro* and in animal data with retarded myelination, impaired brain growth, microglia activation, decreased astrocyte numbers, and increased oligodendrocytes (3). Therefore, exposed infants should be carefully evaluated already during the NICU stay, ruling out the presence of major brain lesions and monitoring the growth of the main cerebral structures (34, 35).

The main limitations of our study are the retrospective single-center design with a small sample of exposed infants, the lack of data about maternal antibiotic treatment administered during pregnancy, the lack of complete data about chorioamnionitis and placental pathology with evidence of inflammation in colonized mother/infant pairs, and the microbial culture initially used to identify these microorganisms in neonates (rather than the current molecular method). Furthermore, infants whose mothers have not been screened were also included in the unexposed group, and some colonized mothers may have been missed due to a single sampling during pregnancy and their infants could have been misclassified.

Finally, we found no significant differences according to the week of GA at diagnosis of *U./M*. spp., probably due to the small sample size of exposed infants. We speculated that, probably, later colonization could have a greater weight in influencing outcomes of preterm infants. To the best of our knowledge, there are no available studies that have yet investigated this aspect.

In conclusion, we analyzed a homogeneous cohort of preterm infants, without significant differences in maternal and neonatal characteristics between the exposed and unexposed groups, in the context of a correct follow-up study. We found that exposed infants to maternal *U./M*. colonization had significantly lower locomotor scores than unexposed peers.

Further prospective multicenter studies in a larger population of preterm infants are needed to better understand if maternal colonization with *Ureaplasma* and *Mycoplasma* could affect neonatal outcomes or not, sparing improper use of antibiotics in mothers and infants.

## Data availability statement

The original contributions presented in this study are included in the article/supplementary material, further inquiries can be directed to the corresponding author.

## Ethics statement

The studies involving human participants were reviewed and approved by Ethics Committee of IRCCS Fondazione Policlinico Universitario “Agostino Gemelli” (Rome, Italy)—in the context of the protocol number 0036181/20 - ID 3244. Written informed consent to participate in this study was provided by the participants’ legal guardian/next of kin.

## Author contributions

FG and DD conceptualized and designed the study, designed the data collection instruments, enrolled subjects, collected the data, analyzed the data, and drafted the initial manuscript. MC, MP, FC, and AB collected the data, analyzed the data, and revised the manuscript. DR, DMR, TS, LM, and EM critically reviewed the manuscript. GV coordinated and supervised the work and critically reviewed the manuscript for important intellectual content. All authors approved the final manuscript as submitted and agreed to be accountable for all the aspects of the work.
